# Electrophysiological biomarkers of behavioral dimensions from cross-species paradigms

**DOI:** 10.1038/s41398-021-01562-w

**Published:** 2021-09-17

**Authors:** James F. Cavanagh, David Gregg, Gregory A. Light, Sarah L. Olguin, Richard F. Sharp, Andrew W. Bismark, Savita G. Bhakta, Neal R. Swerdlow, Jonathan L. Brigman, Jared W. Young

**Affiliations:** 1 Psychology Department, University of New Mexico, Albuquerque, NM USA; 2 Department of Neurosciences, University of New Mexico School of Medicine, Albuquerque, NM 87131 USA; 3 Department of Psychiatry, University of California San Diego, 9500 Gilman Drive MC 0804, La Jolla, CA 92093-0804 USA; 4 VISN-22 Mental Illness Research Education and Clinical Center, VA San Diego Healthcare System, San Diego, CA USA

**Keywords:** Molecular neuroscience, Predictive markers

## Abstract

There has been a fundamental failure to translate preclinically supported research into clinically efficacious treatments for psychiatric disorders. One of the greatest impediments toward improving this species gap has been the difficulty of identifying translatable neurophysiological signals that are related to specific behavioral constructs. Here, we present evidence from three paradigms that were completed by humans and mice using analogous procedures, with each task eliciting candidate a priori defined electrophysiological signals underlying effortful motivation, reinforcement learning, and cognitive control. The effortful motivation was assessed using a progressive ratio breakpoint task, yielding a similar decrease in alpha-band activity over time in both species. Reinforcement learning was assessed via feedback in a probabilistic learning task with delta power significantly modulated by reward surprise in both species. Additionally, cognitive control was assessed in the five-choice continuous performance task, yielding response-locked theta power seen across species, and modulated by difficulty in humans. Together, these successes, and also the teachings from these failures, provide a roadmap towards the use of electrophysiology as a method for translating findings from the preclinical assays to the clinical settings.

## Introduction

Many clinical treatment trials in psychiatry have failed at the cost of time, effort, money, and the hope of the patients tested. These translational failures are often attributed to either a lack of consistent quantification of the same neural processes across species [1, 2] or to the use of “fast and dirty” behavioral techniques that have little-to-no relevance to human testing [3]. In response, the National Institutes of Mental Health (NIMH) formed the Cognitive Neuroscience Treatment Research to Improve Cognition in Schizophrenia (CNTRICS) to identify cognitive systems and component processes that could be tested across species [1]. Continuing this theme, NIMH also initiated the Research Domain Criteria (RDoC) initiative [4, 5], promoting a focus on specific behavioral dimensions and related neurophysiological circuits instead of end phenotypes. A common theme across these new paradigms is the need for brain-based neural signals that are specifically linked to behavioral dimensions, that must be sensitive to systemic alterations due to mental health disorders, and that should ideally be translatable between the species. Ultimately, the availability of specific, sensitive, and translatable neural signals would increase the likelihood of positive animal trial results being translated to positive clinical trial results. Motivated by a specific UH2/3 funding mechanism from the NIMH, we aimed to test three candidate behavioral assays and assess the homology of concurrent neurophysiologic responses across species (UH2 phase), with future studies confirming pharmacologic sensitivity across species (UH3 phase).

Candidate domains that are deficient in psychiatric disorders include effortful motivation, reinforcement learning, and cognitive control. Effortful motivation is recognized as a core contributor to psychosocial impairments in psychiatric conditions, ranging from amotivation in people with schizophrenia and depression to increased goal-directed activity in mania. There are various methods for assessing effort-based decision making, each with associated deficits observed across psychiatric conditions [6–9]. Motivational deficits can also be measured across species, although techniques vary widely [10–12]. One method for measuring effortful motivation is the progressive ratio breakpoint task, linked to a single, well-defined action requirement. Motivation is measured by the point that the participant stops responding to gain a reward, is reduced in people with schizophrenia [13, 14], and accounts for 24% of the variance in their global cognitive functioning [15]. A reduced breakpoint is also observed in animal models relevant to schizophrenia [16], while an increased breakpoint is observed in animal models of mania [17]. Thus, effortful motivation can be measured in a manner consistent across species.

Another promising experimental domain is reinforcement learning, which requires an agent to learn stimulus-action pairings based on rewarding or punishing outcomes. These outcomes are often delivered probabilistically, requiring long-term integration of action values [18, 19]. Probabilistic reinforcement learning paradigms are naturally transferrable across vertebrates [20–23], and are thus an ideal candidate for domain consistency. Probabilistic learning deficits are observed in people with psychiatric conditions, such as schizophrenia [24, 25], bipolar disorder [26], and depression [27–29], bolstering the translational utility of findings. Reinforcement learning theory provides a quantification of abstract processes [30], facilitating an interpretation of neural signals by their confirmation to theorized parameters and computations.

Finally, cognitive control is a domain that is reliably associated with psychiatric distress. Cognitive control requires goal-driven action selection over prepotent tendencies [31, 32], and it can be elicited using several paradigms including various continuous performance tests (CPTs). Prior to the development of the five-choice (5 C)-CPT [33], cognitive control and attention were not typically measurable in the same task in rodents. The 5C-CPT has since been reverse-translated for use in humans and used to provide evidence that cognitive control is deficient in schizophrenia [34] and bipolar disorder [35]. Cross-species pharmacological predictive validity has been demonstrated by the effects of amphetamine, which improves 5C-CPT performance in humans, rats, and mice [35, 36]. Importantly, for cognitive control, a measure of response inhibition (false alarm rate) is functionally separable from the more traditional impulsivity measure of premature responses, as evidenced by dopamine D_4_ receptor and 5-HT_2C_ mechanism sensitivity, respectively [37].

Across these three task domains of effortful motivation, reinforcement learning, and cognitive control it is possible to assess behaviors with preserved consistency across species with outcomes that are sensitive to deficits in clinical populations. However, behavioral consistency has proven insufficient, and shared neural substrates of task engagement are necessary to increase confidence in any treatment translated across species. While there are numerous studies advancing candidate biomarkers of specific domains, many techniques are inherently ill-suited for translating behavioral or neurophysiology between species. Fixed-head techniques like fMRI in humans or calcium imaging in animals have limited translatability. Invasive recordings like depth electrophysiology are compelling but such studies are rare in humans. Electrophysiological recordings naturally encompass multiple scales of measurement in a hierarchical, integrated manner. For example, local fields couple to scalp‐recorded EEG: regardless of scale (depth, dura, scalp, etc.), field activity is always measured [38]. Thus, electrophysiology is uniquely well-suited for addressing questions about translatable neural signal biomarkers.

**Fig. 1:**
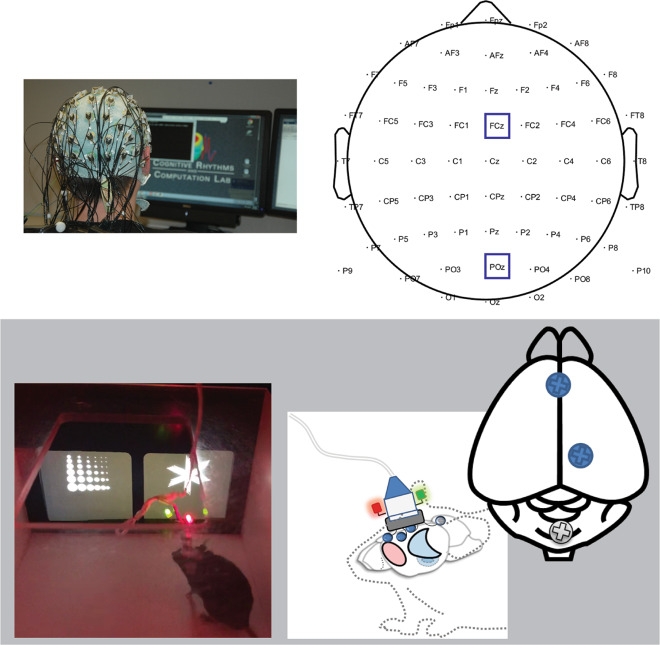
Schematic electroencephalograph (EEG) recording in humans and mice. The present studies utilized EEG recordings in humans and mice while they performed tasks that probed RDoC-relevant domains of functioning, including effortful motivation, reward learning, and cognitive control. Humans used a joystick to respond to on-screen stimuli, while mice responded using a touchscreen. Scalp (human) and dura (mice) EEG recordings were recorded during the execution of these tasks. Time-frequency regions-of-interest were contrasted between task conditions to compare neural signatures of these RDoC domains.

Even with the methodologic promise of comparative electrophysiology, a major impediment toward improving this species gap has been the difficulty of developing paradigms that 1) can quantify EEG responses related to specific behaviors, 2) are impacted by mental health disorders, and 3) are suitable for both human and animal studies. Fortunately, the advent of touchscreen technology for rodents has greatly increased the sophistication of behavioral testing. Here, we detail RDoC-relevant behavioral domains impacted by mental health (effortful motivation, reinforcement learning, and cognitive control) that can be quantified in similar tasks across humans and mice and that are associated with an a priori defined candidate spectral EEG biomarker (Fig. 1). Only some of these behavioral and neural signatures were successfully translated here—yet even failures yielded critical lessons for advancing this field.

## Methods and Materials

### Human participants

The human portion of this study was conducted at the UCSD Medical Center, with approval from the UCSD Human Subject Institutional Review Board. Healthy men and women (18–35 years; *n* = 57) were recruited from the community and monetarily compensated for participation. First, subjects underwent phone screening to assess current and past medical and psychiatric history, medication and recreational drug use, and family history of psychosis. Following informed consent, participants completed an in-depth screening visit, including a physical examination, urine toxicology screen, and urine pregnancy test. All exclusion criteria and data for cohort characterization are presented in the Supplemental Materials. EEG equipment problems with two participants resulted in *n* = 55 participants with available behavioral and EEG recordings across the three tasks.

### Progressive ratio breakpoint task (PRBT)

**Fig. 2:**
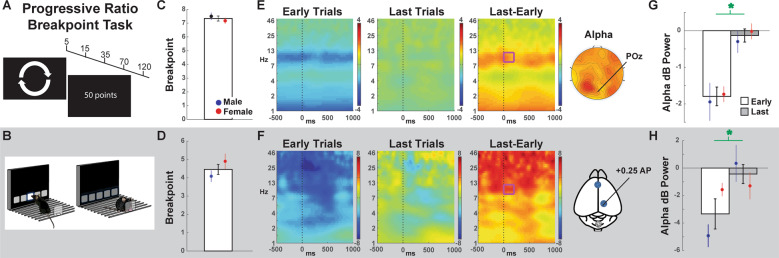
The progressive ratio breakpoint task (PRBT) required the subject to continuously engage in behavior with a diminishing probability of reward. **A** In humans, participants had to rotate a joystick an increasing number of times (e.g. 5, 15, 35…) to accumulate rewards. **B** Mice touched the screen an increasing number of times for the magazine to dispense liquid reward. **C**–**D** Breakpoints for each species including means split by sex. **E**–**F** Time-frequency plots of the earliest vs. the last trials at POz in humans or the posterior lead in mice. For the sake of effective visual comparison, the time dimension is −500 to 1000 locked to markers placed every second (for humans) or every trial (for mice). The magenta box shows the alpha-band tf-ROI. Since the baseline for both species was spread across all trials, all power values are relative (thus “negative” in early trials). **G**–**H** EEG tf-ROI quantification of the early vs. last difference in posterior alpha. Bars indicate the group means (± SEM), green asterisks indicate statistically significant (*p* < 0.05) within-subject differences.

This version of the PRBT has been detailed elsewhere [15] (Fig. 2A). Participants were required to rotate the same arcade joystick handle in the indicated direction to be “rewarded” (50 points/level), with the number of rotations needed set to a progressive ratio schedule (5, 15, 35, 70, 120, etc.). Participants were asked to earn as many points as possible but were told that they could quit any time, ending the entire testing session. A white dot was used as feedback to indicate four successful rotations. The collected “points” held neither value nor were subjects verbally encouraged during task performance. After a short practice session to acclimate to the joystick rotations and task feedback, the test session was initiated. After rotating the joystick a sufficient number of times to attain each reward level, a screen appeared indicating they had earned 50 points and the required direction of rotations alternated (i.e., clockwise to counter-clockwise) to minimize perseverative motor effects. The task ended when patients either completed all possible reward levels, verbally indicated they no longer wanted to continue the task, or failed to make a response for five consecutive minutes. The breakpoint was quantified as the largest number of levels completed before the end of the task.

### Probabilistic learning task (PLT)

**Fig. 3:**
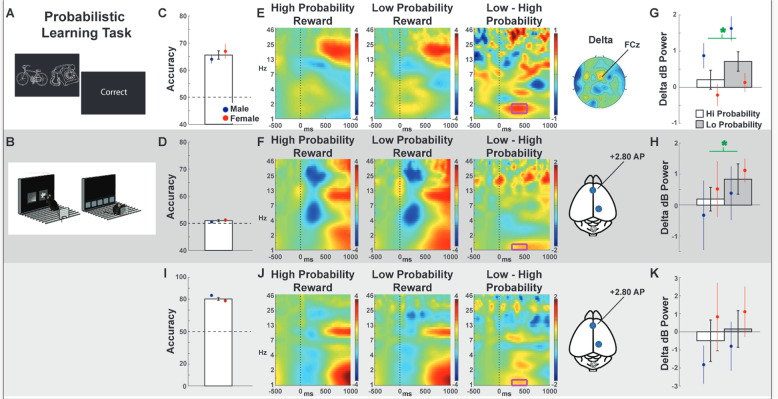
The probabilistic learning task (PLT) required the subject to select the stimulus that probabilistically led to reward most often. **A**–**B** In humans and mice, each trial required a choice between two stimulus icons. **C**–**D** Total accuracies for each species, including means split by sex. **E**–**F** Time-frequency plots of high vs. low probability rewards at FCz in humans or the anterior lead in mice. The magenta box shows the delta-band tf-ROI. **G**–**H** EEG tf-ROI quantification of the difference in reward expectation conditions in frontal delta power. **I**–**K** Replication with a second cohort of mice on a simpler discrimination task. Bars are means (± SEM), green asterisks indicate statistically significant (*p* < 0.05) within-subject differences.

This version of the PLT has also been detailed elsewhere [15] (Fig. 3A). Participants were presented a stimulus pair (e.g., bicycle/phone, chair/clip, plug/flashlight) on a computer monitor and instructed to select the “target” stimulus using a digital four-switch USB arcade-style joystick. Participants were given feedback after each trial about whether their response was “correct” or “incorrect.” Reward probabilities for the target/nontarget stimulus were set within a block of 80 trials (80/20, 70/30, and 60/40), but stimuli differed between trial blocks (first block was bicycle/phone at 80/20, then the next block was chair/clip at 60/40, etc.). Overall performance was calculated as the total number of correct target selections aggregated across the three blocks of 80 trials.

### Five-choice continuous performance task (5C-CPT)

**Fig. 4:**
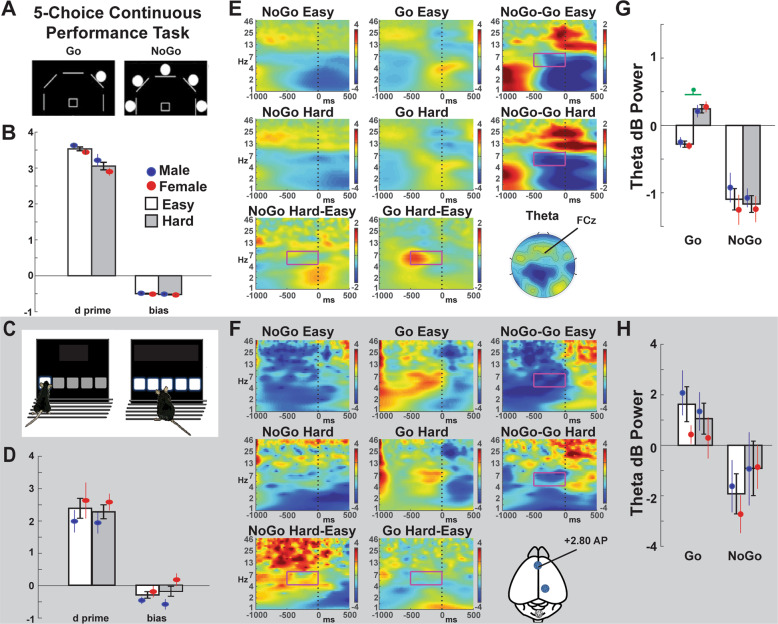
The five-choice continuous performance task (5C-CPT) had two levels of dbifficulty. **A**–**B** In humans, difficulty was manipulated with easy (unmasked) vs. hard (masked) visual contrast conditions. Difficulty altered d prime but not bias. **C**–**D** In mice, difficulty was modulated with easy (3 s delay) vs. hard (1.5 s delay) conditions. Task demand did not change d prime or bias in mice. **E**–**F** Time-frequency plots of response-locked data at FCz in humans or the anterior lead in mice. Since a correct nontarget (nogo) condition does not require a response, these epochs were time-locked to the end of the delay period. The magenta boxes show the theta-band tf-ROI. **G**–**H**) EEG tf-ROI quantification of the go easy vs. go hard difference in preresponse theta power. Green asterisks indicate statistically significant (*p* < 0.05) within-subject differences.

Participants were instructed to move the joystick in the direction that a circle appeared (target stimuli) but inhibit from responding if five circles simultaneously appeared (nontarget stimuli) (Fig. 4A). This new 5C-CPT variant had two different difficulty conditions. In easy conditions, stimuli were presented for 100 ms. In hard conditions, stimuli were presented for 10 ms but then a solid white mask was presented over the stimulus array for 90 ms. All target and nontarget stimuli were presented in a pseudorandom order (to ensure no more than three of the same trial types in a row), with a 1 sec response window available for all trials and a variable intertrial interval (ITI; 500, 1000, or 1500 ms). The full task consisted of 216 trials: 90 target and 18 nontarget stimuli for each of the difficult conditions. Composite metrics of task performance were used in the analysis of performance, including hit rate, false alarm rate (FAR), d prime, and bias.

### Human electrophysiological recording and preprocessing

Continuous electrophysiological (EEG) data were recorded using a BioSemi Active Two system. Data were recorded in DC mode from 64 scalp leads, four electrooculogram (EOG) leads recorded at the superior and inferior orbit of the left eye and outer canthi of each eye, and one nose and two mastoid electrodes for offline re-referencing. The electrode offsets were kept below 25 mV and all channels were referenced to the system’s internal loop (CMS/DRL electrodes). All data were collected using a 512 Hz sampling rate utilizing a first-order antialiasing filter. Custom Matlab scripts and EEGLab [39] functions were used for all data processing. Data were first epoched around the imperative stimuli and then average referenced. Bad channels and bad epochs were identified using a conjunction of the FASTER algorithm [40] and pop_rejchan from EEGLab and were subsequently interpolated and rejected, respectively. Eye blinks were removed following independent component analysis in EEGLab.

### Animal subjects

Male and female C57BL/6 J mice were obtained from The Jackson Laboratory (Bar Harbor, ME), housed in same-sex groupings of two per cage in a temperature- and humidity-controlled vivarium under a reverse 12 h light/dark cycle (lights off:0800 h) and tested during the dark phase. A total of 12 male and 12 female mice were used. All experimental procedures were performed in accordance with the National Institutes of Health Guide for Care and Use of Laboratory Animals and were approved by the University of New Mexico Health Sciences Center Institutional Animal Care and Use Committee. See Supplemental Materials for information on touchscreen pretraining. All rewarding outcomes included the delivery of an auditory tone signaling the subsequent availability of strawberry milkshake.

### Mouse progressive ratio breakpoint task (PRBT)

During the PRBT, mice were presented with a single illuminated square in the center of the touchscreen, which produced a strawberry milkshake reward (40 µL) when pressed. The stimulus remained on the screen until the required response number was made. Each session lasted 60 min. The number of touches required for a reward increased by a step every three trials (e.g.: 1,1,1,2,2,2,4,4,4,7,7,7, etc.). The breakpoint was the last ratio completed at the end of the 1-h session. Mice completed one session of PRBT.

### Mouse probabilistic learning task (PLT)

Throughout each session of the PLT, mice were presented with three pairs of unique stimuli (fan/marble, honey/cave, spider/fan) in three separate 20-trial blocks. For the first block, one stimulus was rewarded 90% of the time and the other was rewarded 10% of the time. The next blocks included 80/20 and then 70/30 reinforcement rates. The mice were given two hours to complete the task. Mice were tested for 1–10 consecutive sessions.

### Mouse five-choice continuous performance task (5C-CPT)

Mice were trained in the 5C-CPT as previously described [36] (see Supplemental Materials and Supplemental Figure S1). Target trials were indicated by illumination of a single stimulus window; nontarget trials consisted of illumination of all five windows. Hits and correct rejections were rewarded. False alarms resulted in a 10 s timeout period. Mice were first trained on a 2:1 ratio (2 target trials to 1 nontarget) for five sessions. They were then tethered to the recording apparatus for two sessions of 2:1 to acclimate to the head stage, and then moved to a 5:1 ratio. Similar to the human 5C-CPT, two different difficulty conditions were included, with easy (3 s response window) and hard (1.5 s response window) trials across ten recording sessions.

### Human and mouse EEG processing

For the sake of descriptive simplicity, both the scalp-recorded signal in humans and the dura-recorded signal in mice are referred to as “EEG.” Time-frequency measures were computed by multiplying the fast Fourier transformed (FFT) power spectrum of single-trial EEG data with the FFT power spectrum of a set of complex Morlet wavelets defined as a Gaussian-windowed complex sine wave: e^i2πtf^e^-t^2/(2xσ^2)^, where *t* is time, *f* is frequency (which increased from 1–50 Hz in 50 logarithmically spaced steps), and the width (or “cycles”) of each frequency band was set to increase from 3/(2πf) to 10/(2πf) as frequency increased. Then, the time series was recovered by computing the inverse FFT. The end result of this process is identical to time-domain signal convolution, and resulted in estimates of instantaneous power taken from the magnitude of the analytic signal. Each epoch was then cut in length (cues: −500 to +1000 ms; responses: -1000 to +500 ms).

Averaged power was normalized by conversion to a decibel (dB) scale (10*log10[power(t)/power(baseline)]), allowing a direct comparison of effects across frequency bands. The baseline consisted of averaged power -300 to -200 ms before all task-specific stimuli, except the response-locked mouse 5C-CPT trials, which benefitted from greater trial-specific clarity by using a preresponse −800 to −700 ms window. A 100 ms duration is often used as an effective baseline, since pixel-wise time-frequency data points have already been resolved over smoothed temporal and frequency dimensions with the wavelets. For the PRBT, the entire duration of all epochs was used as the baseline.

### Statistical analysis

Species were analyzed with separate mixed-effects models. For mice, individual sessions were concatenated and each mouse was treated as a random effect, similar to humans. The contrast conditions within each task were treated as fixed effects. For mouse data, only trials with at least 30 epochs were used in the 5C-CPT or PLT (PRBT always used five trials at the beginning and five trials at the end). In the human dataset, there were clear a priori hypotheses and there was more level-2 data (more subjects), so a smaller threshold was used for level-1 rejection (trials). For the 5C-CPT, this minimum was ten trials and for the PLT, the minimum was 20 trials. For the PRBT, 1-s epochs were averaged for the first 50 s and the last 50 s of the task.

Analysis of Variance (ANOVAs) and *t* tests were used to test hypotheses about condition-specific differences within each task, separately for each species. All tests were two-tailed. We also determined whether sex moderated these effects, although there were no specific hypotheses about the role of sex. Test statistics are shown in Tables 1 & 2. Simple effects contrasts are shown in Table 3 along with the time and frequency ranges for each tf-ROI. All effect sizes are presented as partial eta-squared (_p_η^2^) or Cohen’s *d* (mean difference divided by the pooled standard deviation).

**Table 1:** Test statistics for two (sex) * two (condition) ANOVAs for EEG time-frequency regions-of-interest (tf-ROIs).

PRBT	df	Main: time	Main: sex	Time* sex	PLT	df	Main: probability	Main: sex	Prob* sex
Human	1.50	***F*** = **36.40**, ***p*** **<** **0.001**, _**p**_**η**^**2**^ **=** **0.42**	*F* = 0.45, *p* = 0.50, _p_η^2^ = 0.00	*F* = 0.02, *p* = 0.90, _p_η^2^ = 0.00	Human	1.16	***F*** = 6.67, ***p*** = **0.02**, _**p**_**η**^**2**^ = **0.29**	***F*** **=** 9.25, ***p*** **<** **0.01**, _p_η^2^ **=** **0.37**	*F* = 0.89, *p* = 0.34, _p_η^2^ = 0.05
Mouse	1.18	***F*** **=** 4.44, ***p*** **=** **0.049**, _**p**_**η**^**2**^ = **0.20**	*F* = 1.13, *p* = 0.30, _p_η^2^ = 0.06	*F* = 4.16, *p* = 0.06, _p_η^2^ = 0.19	Mouse	1.11	***F*** **=** **7.01**, ***p*** **=** **0.02**, _**p**_**η**^**2**^ **=** **0.39**	*F* = 0.74, *p* = 0.41, _p_η^2^ = 0.06	*F* = 0.05, *p* = 0.83, _p_η^2^ = 0.01
**5C-CPT**	df	Main: response	Main: difficulty	Main: sex	Resp* diff		Resp* sex	Diff* sex	3-way
Human	1.51	***F*** **=** **82.41**, ***p*** **<** **0.001**, _**p**_**η**^**2**^ **=** **0.62**	***F*** = 6.39, ***p*** **=** **0.02**, _**p**_**η**^**2**^ **=** **0.11**	*F* = 0.63, *p* = 0.43, _p_η^2^ = 0.01	***F*** = 9.22, ***p*** = 0.004, _**p**_**η**^**2**^ **=** **0.15**		*F* = 0.73, *p* = 0.38, _p_η^2^ = 0.01	*F* = 0.15, *p* = 0.71, _p_η^2^ = 0.00	*F* = 0.01, *p* = 0.92, _p_η^2^ = 0.00
Mouse	1.9	**F** **=** **19.03**, ***p*** **<** **0.001**, _**p**_**η**^**2**^ **=** **0.68**	*F* = 0.21, *p* = 0.66, _p_η^2^ = 0.02	*F* = 0.73, *p* = 0.42, _p_η^2^ = 0.08	*F* = 0.47, *p* = 0.51, _p_η^2^ = 0.05		*F* = 0.50, *p* =0.50, _p_η^2^ = 0.05	*F* =0.23, *p* = 0.64, _p_η^2^ = 0.03	*F* = 0.01, *p* = 0.91, _p_η^2^ = 0.00

Bold values represent *p* values and effect size..

**Table 2:** Test statistics for 2 (sex) * 2 (condition) ANOVAs for behavioral performance on the PLT and 5 CCPT.

PLT	df	Main: probability	Main: sex	Prob* sex
Human: accuracy	1.51	***F*** = **54.40**, ***p*** < **0.001**, _**p**_**η**^**2**^ = **0.52**	*F* = 0.87, *p* = 0.36, _p_η^2^ = 0.02	*F* = 0.60, *p* = 0.44, _p_η^2^ = 0.01
Mouse: a ccuracy	1.18	*F* = 0.02, *p* = 0.90, _p_η^2^ = 0.00	*F* = 2.24, *p* = 0.15, _p_η^2^ = 0.11	*F* = 0.34, *p* = 0.55, _p_η^2^ = 0.02
**5C-CPT**	df	Main: difficulty	Main: sex	Diff* sex
Human: hit rate	1.53	***F*** = 23.07 ***p*** **<** 0.001 _**p**_**η**^**2**^ **=** **0.31**	*F* = 1.41 *p* = 0.24 _p_η^2^ = 0.03	*F* = 0.28 *p* = 0.60 _p_η^2^ = 0.00
Mouse:h it rate	1.13	*F* = 0.58 *p* = 0.46 _*p*_*η*^*2*^ *=* 0.04	*F* = 0.02 *p* = 0.89 _*p*_*η*^*2*^ *=* 0.00	*F* = 3.08 *p* = 0.10 _*p*_*η*^*2*^ = 0.17
Human: FA	1.53	*F* = 2.01 *p* = 0.16 _*p*_*η*^*2*^ = 0.04	*F* = 0.97 *p* = 0.33 _*p*_η^2^ = *0.02*	*F* = 0.46 *p* = 0.50 _*p*_*η*^*2*^ *=* 0.01
Mouse: FA	1.13	F = 4.33 *p* = *0.06* _*p*_*η*^*2*^ *=* 0.24	*F* = 2.32 *p* = 0.15 _*p*_*η*^*2*^ *=* 0.14	*F* = 0.11 *p* = 0.75 _*p*_η^2^ = *0.01\*
Human: d prime	1.53	***F*** ***=*** **28.78** ***p*** **<** **0.001** _**p**_***η***^**2**^ ***=*** **0.36**	*F* = 3.27 *p* = 0.08 _*p*_η^2^ = *0.06*	*F* = 0.52 *p* = 0.47 _*p*_*η*^*2*^ *=* 0.01
Mouse: d prime	1.13	*F* = 0.91 *p* = 0.34 _*p*_*η*^*2*^ *=* 0.07	*F* = 0.53 *p* = 0.48 _*p*_*η*^*2*^ *=* 0.04	*F* *=* 0.13 *p* = 0.72 _*p*_*η*^*2*^ *=* 0.01
Human: bias	1.53	*F* = 1.33 *p* = 0.25 _*p*_*η*^*2*^ = 0.03	*F* = 1.72 *p* = 0.20 _*p*_*η*^*2*^ *=* 0.03	*F* = 0.0 *p* = 0.99 _*p*_*η*^*2*^ = 0.00
Mouse:b ias	1.13	*F* = 3.48 *p* = 0.09 _*p*_*η*^*2*^ *=* 0.21	*F* = 3.52 *p* = 0.08 _*p*_*η*^*2*^ *=* 0.21	*F* = 1.98 *p* = 0.18 _*p*_*η*^*2*^ = 0.13
Human: hit RT	1.53	***F*** ***=*** **146.59** ***p*** ***<*** ***0.001*** _**p**_***η***^**2**^ ***=*** **0.73**	*F* = 1.76, *p* = 0.19*,*_*p*_*η*^*2*^ *=* 0.03	*F* = 2.80*, p* = 0.10*,*_*p*_*η*^*2*^ *=* 0.05
Mouse: hit RT	1.13	***F*** ***=*** **9.29** ***p*** = **0.008** _**p**_***η***^**2**^ **0.38**	*F* *=* 1.18, *p* = 0.30, _*p*_*η*^*2*^ *=* 0.07	*F* = 1.45, *p* = 0.25, _p_*η*^2^ = 0.09

Bold values represent *p* values and effect sizes.

**Table 3:** Summary of simple effects.

		Low freq	High freq	Start time	End time	*t*	df	*p*	*d*	Match?
PRBT alpha	Human	8	12	0	200	6.14	51	<0.001	0.92	Yes
	Mouse	8	12	0	200	2.15	19	0.04	0.66	
PLT delta	Human	1.3	2	250	550	2.44	17	0.03	0.56	Yes
	Mouse	1	1.4	250	550	2.78	12	0.02	0.40	
5C-CPT theta	Human	4	8	−500	0	5.58	54	<0.001	1.06	No
	Mouse	4	8	−500	0	0.68	10	0.51	0.26	

Time and frequency ranges for event-related tf-ROIs and simple effects statistical contrasts (paired *t* statistic, Cohen’s *d*) within each task. For the PRBT, the contrasts are early > late trials. For PLT, contrasts are low > high reward probability. For the 5C-CPT, the simple effect is the go hard > go easy condition.

## Results

Statistical differentiation followed an a priori approach, where each task had a predicted spatial, temporal, and frequency range for the contrast of interest. These time-frequency regions-of-Interest (tf-ROIs) were broadly defined based on well-replicated findings from the human EEG literature (detailed for each task below). In the discussion, we note how the exact tf-ROIs discovered here will be used in future pharmacologic studies, providing a chance for direct replication and theoretical extension of the candidate biomarkers. Each figure shows the tf-ROI in magenta, as well as topographic plots highlighting the target electrode.

### Predictions: PRBT

This task required subjects to engage in active behavior to gain a reward at each level. In humans, levels increased after rotating the joystick, while in mice, levels increased after sufficient touches to the screen. In both cases, the number of actions required for the next reward progressively increased. The point at which the subject stopped responding was identified as their breakpoint and was used as an index of effortful motivation. Previous EEG studies have implicated alpha power as a concomitant of effortful behavior in humans [41–43], including changes due to physical and mental fatigue [44, 45]. Here, we examined if this relationship was present during the PRBT and if it was common between species. The alpha-band was defined as 8–12 Hz, and electrode POz was selected to be within the mass of broad posterior alpha. Epochs were locked to the first 50 and last 50 s at electrode POz in humans, and to the first five and last five rewarded responses in the posterior lead in rodents. Since this alpha-band effect was expected to be relatively consistent across events, the time window was arbitrarily set from 0–200 ms postevent. It was hypothesized that alpha power at this posterior lead would be larger at the end of the task, as indicated for physical vs. cognitive effort [46].

### Outcomes: PRBT

In humans, the breakpoint was around 7 (Fig. 2C). In mice, the breakpoint was around 4 (Fig. 2D). There were no sex differences in either the number of trials completed or the breakpoint (human *t’s* < 1, mouse *t’s* < 1.52). Following minimum epoch count requirements, and due to two technical problems in human EEG, there were *n* = 52 humans (*M* = 24, *F* = 28) and *n* = 20 mice (*M* = 11, *F* = 9). Both the humans and mice had a significant late > early alpha power contrast (Table 1). There were no main or interactive effects with sex for either species.

Unlike the other experiments in this report, and to the best of our knowledge, the hypothesis of an alpha-band marker of breakpoint-related effort had not been tested. This alpha difference (last minus first) was proposed to scale with greater motivation loss, and it was indeed negatively correlated with the breakpoint in humans (*ρ* (52) = −0.28, *p* = 0.046; Supplemental Figure S2). Notably, time-on-task, as measured by the number of seconds on the PRBT did not correlate with breakpoint (rho(52) = −0.15, *p* = 0.30). This outcome highlights the fact that participants achieved a higher breakpoint through effort, which correlated with alpha-band difference, not time. A stepwise regression verified this specific relationship, where seconds did not correlate with the alpha difference (*F* < 1), yet the addition of the breakpoint in the next level led to a significant *F* change (*F*(2,49) = 4.03, *p* = 0.02, *R*^2^ change = 0.10). The analysis of mouse performance required some different operational definitions and statistical approaches, since they always had one hour to complete the task and most mice stopped at a breakpoint of “four” while a few stopped at “seven.” In mice, there was no relationship between alpha power and the number of epochs completed (rho(22) = −0.09, *p* = 0.70), although this may be due to a reduced sample size. When analyzed as two groups, the mice with a breakpoint of “four” had a nonsignificantly higher alpha power than those with a breakpoint of “seven” (*t*(18) = 1.21, *p* = 0.24), supporting the premise that a higher sample size may have yielded the same correlation seen in humans.

### Predictions: PLT

Trials that resulted in correct feedbacks were used for all analyses. In mice, rewarded responses were immediately indicated by a 1 s, pure noise tone concomitant with the illumination of the magazine light and delivery of the reward. Comparisons were split based on the probabilistic aspect of the reward feedback, creating high probability (i.e., target response followed by reward) vs. low probability (i.e., nontarget response followed by reward) contrasts. While this contrast is ideal for comparing the same process without interference from different sensory or imperative events, it unfortunately conflicted with our strong epoch count requirements (see Methods and Materials). These criteria led to the necessity of limiting these analyses to only the humans and mice who experienced the minimum amount of both trial types. Epochs were locked to rewarding feedbacks at electrode FCz in humans—where the reward positivity ERP component is maximal [47–49]—and at the frontal lead in rodents. We hypothesized that low vs. high probability rewards would elicit a frontal midline delta-band power burst [47, 50]. While this reward-locked delta burst is reliably observed in humans, the timing and frequency varies between the published studies [47, 49–51]. Here, the temporal window was defined from 250 to 550 ms post-feedback; however, the frequency window was 1.3–2 Hz for humans and 1–1.4 Hz for mice.

### Outcomes: PLT

For humans, overall PLT accuracy was greater than chance, with no difference between the sexes (Table 2). For mice, overall accuracy did not differ from chance. However, many mice were excluded from subsequent analysis due to a low number of epochs; the accuracy of the cohort used in EEG analysis was significantly higher than chance (*t*(13) = 2.26, *p* = 0.04, *d* = 0.60), with no difference between sexes (*t* < 1). Following these minimum epoch requirements for high and low probability events, the sample sizes of EEG analyses were reduced (human: *M* = 7, *F* = 11; mouse: *M* = 5, *F* = 8). Both the humans and mice had a significant low > high probability delta-band contrast, with a significant main effect of sex in humans (males > female), (Table 1).

While this carefully contrasted delta-band effect in mice is compelling, it was disappointing that the mice performed so indiscriminately during EEG assessment. To test the reliability of this delta-band contrast, a separate cohort (*N* = 12: *M* = 6, *F* = 6) was tested over g days on a single pair of stimuli that had 100 vs. 50% probabilities of reward. All mice performed at around 80% accuracy (i.e., they selected the 100% rewarding option 80% of the time: *t*(11) = 20.90, *p* < 0.001, *d* = 6.03), suggesting a high level of intrinsic exploration (Fig. 3I). Critically, time-frequency contrasts revealed a surprise-evoked delta-band burst in the same tf-ROI (Fig. 3J-K). Although this cohort did not reveal a significant statistical differentiation between conditions (*t*(11) = 0.89, *p* = 0.39, *d* = 0.18), this may still be expected from a true effect. The *p*-value alone is a poor metric for assessing replicability; effect sizes and confidence intervals are more useful for assessing the utility of an experimental outcome [52, 53]. Here, we observed that the mean difference between conditions were in fact the exact same number (first cohort: mean difference = 0.65 dB, CI = 0.14, 1.15; second cohort mean difference = 0.65 dB, CI = −0.97, 2.27). Although not included in the a priori hypotheses, analyses for EEG time-frequency region of interests for punishment-related theta with statistical analyses (Supplemental Tables S1 & S2), with corresponding theta power representation (Supplemental Figure S3), are described, in addition to correlations to mouse accuracy related to reward- and punishment-associated delta power differences (Supplemental Figure S4).

### Predictions: 5C-CPT

Only hits on target trials and correct rejections on nontarget trials were used for EEG analysis. This novel 5C-CPT also introduced two varying difficulty levels using backward masks. In humans, these were easy (standard, unmasked) and hard (masked) visual contrast conditions. In rodents, we utilized supposedly easy (3 s delay) and hard (1.5 s delay) conditions. In mice, rewards were immediately indicated by a 1 s, pure noise tone concomitant with the illumination of the magazine light and delivery of reward. These rewards were locked to the response on hits and the end of the delay period on correct rejections. The nontarget vs. target contrasts were expected to elicit frontal midline theta power, which is a reliable indicator of cognitive conflict [54, 55]. However, it was not possible to verify that cues were visually attended to by the mice, so response-locked epochs were used for both species. Epochs were locked to responses at electrode FCz in humans and the frontal lead in rodents. Since there were no responses for correct rejections, nontarget trials were time-locked to the end of the temporal epoch. The theta-band was defined as 4–8 Hz. Since conflict-specific theta power peaks at FCz before response execution [56, 57], the temporal window was defined as −500 to 0 ms preresponse. This frontal theta effect was hypothesized to be parametrically enhanced in the hard vs. easy contrast.

### Outcomes: 5C-CPT

In humans, the difficulty manipulation (masking), caused a significantly lower hit rate, longer RTs, and lower d prime, indicative of worse attention but no change to false alarms (response inhibition) or, importantly, bias of responding. There were no main or interactive effects with sex, (Table 2). In mice, the difficulty manipulation (stimulus duration), induced faster RTs but no changes to performance measures. There were no main or interactive effects with sex (Table 2). Following minimum epoch count requirements, there were *n* = 55 humans (*M* = 26, *F* = 29) and *n* = 11 mice (*M* = 8, *F* = 3). In humans, there were significant main effects of preresponse theta power to response (target > nontarget) and difficulty (hard > easy), and an interaction (hard target > easy target > nontarget) (Table 1). For mice, there was only a significant main effect of response (target > nontarget). All other *F* tests < 1 (Table 1). Since the response data were locked to different events (there were no responses on nontarget trials), this response contrast was not an effective assessment of cognitive control, more likely reflecting attentive functioning. The contrast between difficulty conditions is better-suited as an assessment of control since the imperative events were identical. Preresponse theta was only modulated by difficulty in humans (hard target > easy target), while there was no effect in mice (Table 3). There were no main or interactive effects with sex for either species (Tables 2 and 3).

## Discussion

Here, we report that consistent behaviors and related neural signatures can be elicited across various tasks and domains in humans and mice. These candidate EEG responses displayed remarkable temporal, spatial, and frequency consistency between species, largely consistent with our a prior hypotheses. Specifically, the PRBT (effortful motivation) and PLT (reward learning) revealed consistent neural signatures of posterior alpha and reward delta respectively, seen in both humans and mice while performing these tasks. Additionally, the 5C-CPT revealed consistent target-locked theta across species.

### Effortful motivation: PRBT

The behavioral performance of humans and mice in the PRBT was consistent with earlier reports [15, 16, 58]. Previous EEG studies have implicated alpha power with effortful behavior in humans [41–43], including changes due to physical and mental fatigue [44, 45]. More recently, diminished alpha power was described in mice lacking metabotropic glutamate receptor 5 [59], and rats lacking the Fmr1 gene [60], although it is not clear if this was tied to motivational state since it was simply in awake rodents. Our present data, therefore, add to human literature showing a duration-specific decline in posterior alpha power in humans, confirming this same effect in mice performing the PRBT, thereby enabling assessment of both patient populations and their rodent models. The scale of this alpha power decline correlated with the breakpoint in humans, but evidence for a similar relationship in mice was uncertain, likely due to lower sample sizes. Some evidence in support of the relationship emerged when comparing the alpha power of animals with differing breakpoints and requires future study. Given that posterior alpha is the single most dominant background rhythm in humans, these data support the idea that some common neural architecture is preserved across mammalian species that is stimulated during the performance of the same task. Future studies will have to confirm that this neural correlate of effortful performance is altered across clinical populations and in animals manipulated to be relevant to the population, and whether it is sensitive to pharmacologic agents.

### Reward learning: PLT

While humans were predictably effective at performing this task, mice performed just above chance, unless the task was simplified. Despite these addressable difficulties in training and performance, the similarities between tasks facilitates comparison of EEG responses during task completion. The analytic contrasts were able to be well-controlled within each species, facilitating a comparison of the underlying process (e.g., low vs. high probability corresponding to high vs. low reinforcement prediction error), without interference from different sensory or imperative stimuli. The prediction of a delta-band enhancement to reward surprise was borne out in both species. An additional study with easier discriminability replicated the observation of the delta-band effect with consistent confidence intervals, albeit not the statistical differentiation. This spectral representation of the reward positivity ERP component has been described in humans, particularly its sensitivity to formal estimates of reward prediction error [50]. These findings are the first demonstration of this same spectral response in dura-recording from rodents, although a similar slow cingulate-recorded ERP response in this same time range was observed in the difference between the reward and punishment trials in rats [61]. Mice are a prey species and are more sensitive to punishment [62, 63] than rats in similar paradigms [64]. Although not specified by our a priori predictions, we also investigated punishment surprise-evoked theta power (Supplemental Figure S4). However, this response was not significantly modulated in mice.

### Cognitive control: 5C-CPT

The 5C-CPT assesses cognitive control and is sensitive to deficits in clinical populations and modulations by pharmacologic agents. Although humans easily maintain focus on the screen between stimuli (enabling EEG assessment locked to stimulus presentation), such assessment is much more difficult in mice given their need to turn around toward the food delivery area, thereby increasing misses to the moment of stimulus presentation, limiting stimulus-locked EEG events. Without aggressive implementation changes, such as head-fixing, mice are unlikely to reliably visually attend to the screen during the ITI, driving stimulus-locked EEG events, unlike humans. The addition of different auditory tones for target and nontarget trials may be needed for effective stimulus-locked manipulation for future trials, though the need for trial-and-error parameterization will likely delay the utilization of this task. The response-locked differentiation of EEG signals to target and nontarget trials presented here is technically a misnomer because correct rejections to nontarget trials do not include a response. These EEG “responses” were at the end of the hold period, thus, the intrinsic EEG response differed between conditions, by definition. The novel difficulty manipulation was, therefore, used to assess related domain constructs on hit trials where the imperative event (i.e., a response to targets) was identical.

Response-locked theta was strongly enhanced in more difficult hit trials in humans. While response-locked theta was seen in mice, no effect of difficulty was observed on performance or this EEG response in mice. This difference likely reflects the ineffectiveness of manipulating trial difficulty based on stimulus durations by trial type in mice—shorter delays make target trials more difficult but makes withholding from nontarget trials easier. Ultimately, more work is required for manipulation of spatial attention and parameterization of difficulty in mice (e.g., a similar backward mask used in humans), although the addition of discriminant auditory tones may be able to address multiple issues. A wealth of prior findings suggests that it is too early to rule out frontal theta as a viable candidate for cross-species translation. Posterror cingulate theta power enhancement has been shown in humans and rats [65], as has a cue-locked dopamine-dependent theta signal [66]. These data, therefore, provide support but require further work.

### Limitations and future directions

While the mere concept of comparing cross-species brain responses deserves a critical appraisal, there is good reason to theorize that some electrophysiological activities remain preserved across species. Although classic EEG frequencies are non-specifically related to cognitive constructs and are likely to simply reflect the intrinsic computations of the generative cortex, event-related local field oscillations are closely linked to any neuronal mechanism that implements neural computations [67–70]. There is a marked preservation of temporal activity across vertebrate brains, likely due to architectural adjustments that evolved to prioritize retention of temporal coding schemes [71]. Increasing evidence also confirms neurodevelopmental CNS synchronization in EEG responses between humans and rodents, as well as the consistent impact of alcohol and auditory stimuli on these event-related oscillations [65, 72–74]. These theoretical justifications and empirical outcomes are compelling, and they dovetail with the potential for assessing electrophysiology in each species.

Statistical effects reported here were modest. As noted earlier, modulation of these exact tf-ROIs will be tested in future studies as a continuation of the novel UH funding mechanism via an overall “learn-confirm” design strategy. This report serves to convey a crystallized set of parameters that will be used in future tests of pharmacologic modulation. With additional experiments and increased sample sizes in mice (comparable to that of humans), the degree of test-retest reliability will be established and further consistencies may be revealed across species. We included both males and females of both species and, while sex differences in learning have been reported [75–77], we largely have not seen such sex differences. These future studies will add to our current knowledge.

These data only compared findings from a single electrode in humans with a single dura lead in mice. While this theory-driven reduction of spatial dimensionality is appropriate with our a priori hypotheses and the preliminary goals of this study, it offers only a fraction of assessable EEG activities in each species. Any conclusion of translational similarity is also based on a qualitative assessment of common within-species statistical effects. While this simplicity is beneficial here, future comparative studies could utilize data normalization, computational modeling, and covariance statistics for quantitative assessments of common neural signatures between species. Notably, these data-driven strategies require a large amount of data, and thus they are not likely to be undertaken unless they follow compelling findings from small-scale hypothesis-driven studies, as presented here.

## Conclusion

The failure of preclinical models based on behavioral measures alone is well-established. If we are to understand the complex neural mechanisms underlying cognitive deficits in psychiatric disorders, novel approaches linked to neural outcomes must be taken. This field is most likely to advance by investigating similar bio-signals between species. The comparison of mouse and human event-related EEG responses is, therefore, an appropriate next step, based not only on the methodological advantages but also the theoretical similarities between potentially preserved neural mechanisms. Here, we present three tasks that are for the first time revealing a common translational event-related EEG responses between humans and mice.

Importantly, the PRBT revealed that arousal-related posterior alpha appears common between species, and it should be easy to assess the generalizability of this effect within a variety of other tasks. From the PLT, we reveal a very compelling similarity between species based on a common computation defined by reinforcement learning: the degree of reward surprise coded within mid-frontal delta-band power. These two successful paradigms—PLT and PRBT—are both currently being assessed with pharmacologic manipulations across species. While the 5C-CPT presented potential consistencies with target-locked theta seen across species, more work is required for parametric confirmation in mice. The candidate biomarkers advanced here will soon be further evaluated as electrophysiological signatures of behavioral dimensions from cross-species paradigms.

## Supplementary information

Supplemental Material

## Data Availability

All data and Matlab codes are available on Openneuro.org, accession #ds003638.
