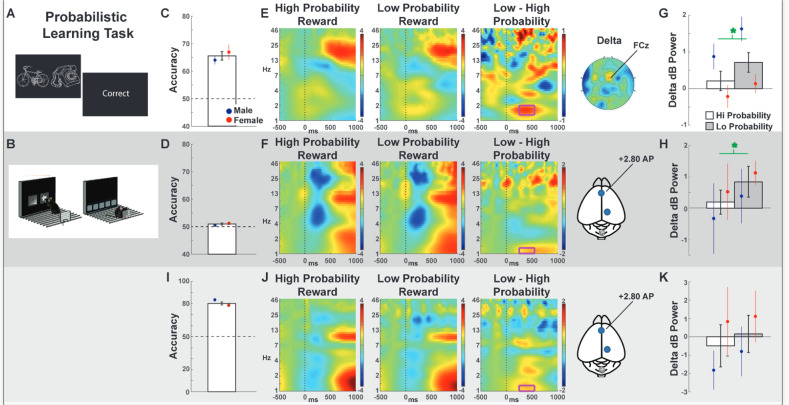# Correction to: Electrophysiological biomarkers of behavioral dimensions from cross-species paradigms

**DOI:** 10.1038/s41398-022-02070-1

**Published:** 2022-07-21

**Authors:** James F. Cavanagh, David Gregg, Gregory A. Light, Sarah L. Olguin, Richard F. Sharp, Andrew W. Bismark, Savita G. Bhakta, Neal R. Swerdlow, Jonathan L. Brigman, Jared W. Young

**Affiliations:** 1 Psychology Department, University of New Mexico, Albuquerque, NM USA; 2 Department of Neurosciences, University of New Mexico School of Medicine, Albuquerque, NM 87131 USA; 3 Department of Psychiatry, University of California San Diego, 9500 Gilman Drive MC 0804, La Jolla, CA 92093-0804 USA; 4 VISN-22 Mental Illness Research Education and Clinical Center, VA San Diego Healthcare System, San Diego, CA USA

**Keywords:** Molecular neuroscience, Predictive markers

Correction to: *Translational Psychiatry* 10.1038/s41398-021-01562-w, published online 17 September 2021

The original version of this article unfortunately contained a mistake. The correction is to swap the labels for figure 3, representing Lo and Hi Probability in panels G, H, and K. The corrected figure can be found below. The original article has been corrected.