# Supplementary material for: Electrophysiological biomarkers of behavioral dimensions from cross-species paradigms
Source: Transl Psychiatry. 2021 Sep 17;11:482. doi: 10.1038/s41398-021-01562-w (PMC8448772; doi:10.1038/s41398-021-01562-w)
Supplement: Supplementary file 1 — Supplemental Material [file 41398_2021_1562_MOESM1_ESM.docx]

**Electrophysiological biomarkers of behavioral dimensions**

**from cross-species paradigms**

James F. Cavanagh, David Gregg, Gregory A. Light, Sarah Olguin, Richard F. Sharp, Andrew W. Bismark, Savita G. Bhakta, Neal R. Swerdlow, Jonathan L. Brigman, Jared W. Young

SUPPLEMENTAL MATERIALS

**Methods and Materials**

*Human Participants: Exclusions*

Age: < 18 or > 50

Medical: History of serious medical illness, e.g., insulin-dependent diabetes, HIV, AIDS, cancer, stroke, heart attack, hypertension, uncontrolled hypothyroidism, seizure disorder, head injury with sustained loss of consciousness, sleep apnea; physical examination findings: focal or generalized neurological findings, evidence of cardiopulmonary disease, hepatomegaly, splenomegaly or abdominal mass; electrocardiogram abnormalities; injury to dominant hand; positive pregnancy test (urine).

Medications: Current or lifetime use of prescribed psychotropic medications; current use of diet pills or over-the-counter medications that contain phenylephrine, diphenhydramine, St. John’s Wort, Milk Thistle.

Substance: Self-report of any illicit drug use within the last 30 days; positive urine toxicology for illicit drugs; self-report of lifetime use of hallucinogens, phencyclidine, intravenous drugs; treatment received for drug or alcohol use disorder.

Psychiatric: current or lifetime history of major mental illness. Family history of psychotic disorder, bipolar disorder or obsessive compulsive disorder in a first-degree relative.

*Animal Subjects: Pre-Training*

All operant behavior was conducted in a custom acrylic chamber measuring 21.6 × 17.8 × 12.7 cm housed within a sound- and light-attenuating box (Med Associates, St. Albans, VT) as previously described^76^. One end of the chamber contained a house light, a tone generator, an ultra-sensitive lever, and a liquid dispenser delivering strawberry milk liquid reward (Nesquik, S.A., Vevey, Switzerland), Carnation powdered milk (Nestle Baking, a division of Nestle USA, Inc, Solon, OH, USA), and water) into a reward magazine. The other end of the chamber was fit with a touch-sensitive screen (Conclusive Solutions, Sawbridgeworth, UK) covered by a black acrylic aperture plate allowing two or five active touch areas measuring 2.5 × 2.5 cm separated by 0.6 cm and located at a height of 1.6 cm from the floor. Stimulus presentation in the response windows and touches were controlled and recorded by the K-Limbic Software Package (Conclusive Solutions, Sawbridgeworth, UK).

The weights of mice were first slowly reduced and then maintained at 85% free-feeding body weight. Prior to training, mice were acclimated to the strawberry milk reward by providing 3 mL of reward per mouse on a weigh boat in the home cage for three days. After becoming acclimated to the reward, mice were habituated to the operant chamber and the magazine with 30 min sessions where 40 µL of strawberry milk was dispensed 60 sec after each collection (measured by a head dip into the magazine). Mice that retrieved 10 rewards within 15 min were moved to touch training. A session during training consisted of 30 total trials or 30 min, whichever was achieved first. Touch training required mice to first initiate each trial with a nose poke into the magazine, followed by a 4 sec ITI, and then respond to a presentation of a white square stimulus in one of the five response windows (spatially pseudorandomized). The stimulus remained on the screen until a response was made to the stimulus. These responses resulted in reward presentation, a 1 sec tone, and illumination of the magazine.

Once the mouse finished drinking and withdrew, the magazine light was re-illuminated for initiation of the next trial. Mice that retrieved 30 rewards within 30 min were moved to “punish” training where incorrect responses resulted in a 10 sec timeout with the house light on to discourage indiscriminate responding. Mice could also respond during the ITI, but this was discouraged with a 10 sec timeout with the house light on. Mice that initiated 30 touches within 30 minutes were then moved to standard training. In this phase, the stimulus remained on the screen for a limited amount of time followed by a 2 sec hold where the touch windows remained active. This phase was broken up into five steps with stimulus durations of 20 sec, 10 sec, 8 sec, 4 sec, and 2 sec. A mouse moved to the next step after achieving two consecutive days with a mean correct latency of ≤ half of the stimulus duration (defined as the time between when stimulus was presented to when a response was made to the illuminated stimulus). After completion of the final step mice were given *ad libitum* food for three days prior to surgery.

In surgery, mice were anesthetized with isoflurane and placed in a stereotaxic alignment system (Kopf Instruments, Tujunga, CA) for fitting with dura-resting leads. Skull screws were 0.078” stainless steel machine screws (00.90-078-M-SS-PS US Micro Screw) that were placed targeting medial prefrontal cortex (mPFC: AP +2.80, ML +0.00) and posterior parietal association cortex (PtA: AP -1.46, ML +1.00) with a cerebellar ground. Silver wire leads were soldered to the pins of Omnetics connectors, wrapped securely around each corresponding screw, and secured to the skull using dental cement. After seven days of recovery, body weight reduction resumed and mice were given a post-surgery reminder session consisting of the last pre-training regimen to ensure retention of pre-training criterion. During each recording session, electrophysiological activity was captured via a multichannel acquisition processor (PlexControl, Plexon) at a sample rate of 1000 Hz and behavior automatically video-tracked via head stage-integrated LED to exclude trial irrelevant behavior (CinePlex, Plexon). Relevant task events and behaviors were time-coded as event markers on the recording file via TTL pulse from the behavioral software, and were confirmed via video tracking.

*Mouse 5C-CPT*

Following touchscreen training, mice were trained on the 5-choice serial-reaction time (5C-SRT) task which required mice to touch a stimulus that appeared briefly for 20 sec followed by a 2 sec limited hold (square is no long illuminated, but response can still be made). Stimulus duration was reduced to 10 sec, 8 sec, 4 sec, and 2 sec as criterion was met or six sessions were completed. Lastly, mice completed a 2 sec stimulus duration with a variable ITI of 3-7 sec. Criterion was ≥30% correct responses over a 120-trial session and a mean correct latency of half the stimulus duration for two consecutive sessions. Following 5C-SRT training, mice were trained on the 5C-CPT by the addition of non-target (hold) trials.

**Results**

*Mouse 5CSRT Task Pre-Training*

No significant differences by sex were seen on the number of sessions required to learn initiation and responding to one continuously presented stimulus on the touchscreen for reward (F(1,24)=1.77, *p*=0.19). When animals were tested on the 5CSRT task, all mice showed reduced hit rate as stimulus duration decreased (e.g., 20, 10, 8, 4, & 2 sec) but groups did not significantly differ by sex (duration: F(5,120)=77.75, *p*=0.01; sex: F(1,24)=1.09, *p*=0.30). There was a significant sex x duration interaction (F(5,120)=3.54, *p*=0.01). Similarly, all mice required more sessions to reach criteria as the duration of stimulus presentation decreased (F(1,120)=9.74). Similarly, no significant differences on sessions to reach criterion were seen between sex (F(1,24)=0.77, *p*=0.39), but there was a significant sex x duration interaction (F(5,120)=3.48, *p*=0.01). As stimulus duration decreased, mean correct latency also significantly decreased across groups (F(1,120)=225.28, *p*=0.01) and there was a significant main effect of sex (F(1,24)=7.51, *p*=0.01) and a sex x duration interaction (F(5,120)=0.73, *p*=0.03). Premature responses also significantly increased across groups as stimulus durations decreased (F(1,120)=10.94, *p*=0.01) and significantly differed by sex (F(1,24)=5.44, *p*=0.03] with no interaction. Trials omitted also increased significantly when stimulus duration decreased (F(1,120)=158.80, *p*=0.01) with no main effect of sex ( F(1,24)=0.04, *p*=0.83;] but there was a sex x duration interaction (F(5,120)=5.45 *p*=0.01). Finally, magazine latency, a measure of motivation to retrieve reward, did not significantly vary with duration (F_(1,120)_=1.90, *p*=0.09) or sex (F_(1,24)_=0.01, *p*=0.97) with no interaction.


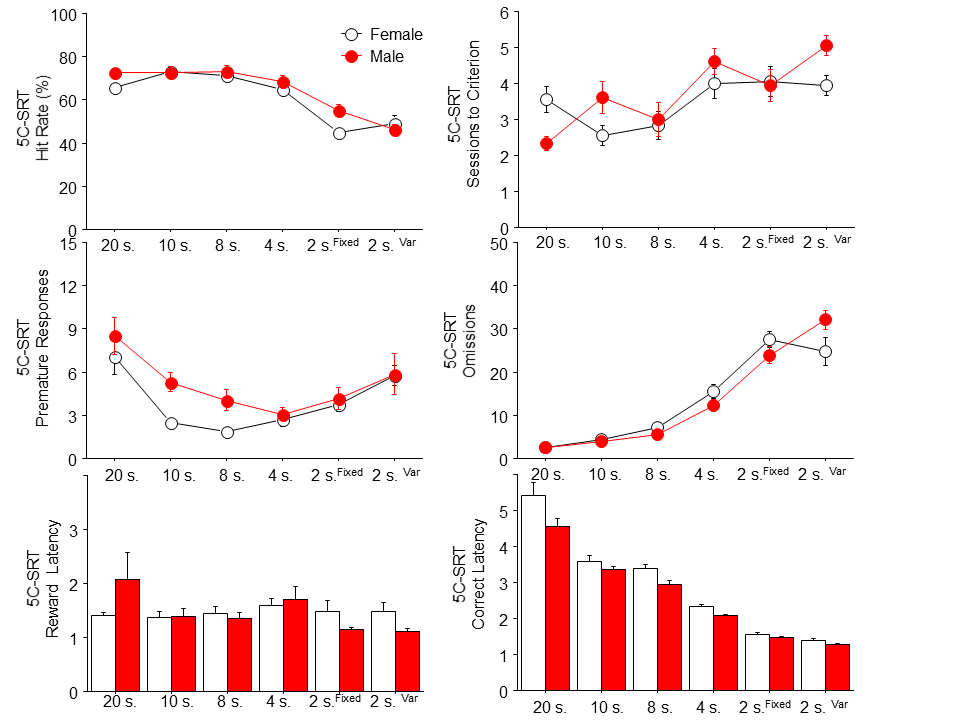


**Figure S1.** **Mouse pre-training performance on the 5C-SRT task at varying response latency criteria.**

*PRBT Neural Signals: posterior alpha power linked to behavioral outcome*

| **PLT** | df | Main: Probability | Main: Sex | Prob*Sex |
| --- | --- | --- | --- | --- |
| Human: Punishment | 1,30 | **F=5.79, *p*=0.02, _p_η^2^=0.16** | F=1.34, *p*=0.26, _p_η^2^=0.04 | F=3.06, *p*=0.09, _p_η^2^=0.09 |
| Mouse:  Punishment | 1,12 | F=0.31, *p*=0.59, _p_η^2^=0.03 | **F=6.77, *p*=0.02, _p_η^2^=0.36** | F=1.88, *p*=0.20, _p_η^2^=0.14 |

**Table S1.** **Test statistics for 2 (sex) * 2 (condition) ANOVAs for EEG time-frequency Regions-of-Interest (tf-ROIs).**

Supplemental Figure S2 details that the breakpoint of humans in the PRBT revealed a negative correlation with posterior alpha power.

*PLT Neural Signals: Punishment Theta Power*

Punished responses were immediately followed by the illumination of the house light for 10 sec. Comparisons were split based on the probabilistic aspect of the punishment feedback, creating high probability (i.e., non-target response followed by punishment) vs. low probability (i.e., target response followed by punishment) contrasts. Low vs. high probability punishment contrasts were proposed to elicit a frontal midline theta-band power burst observed in humans^77^. Following minimum epoch requirements for high and low probability events, sample sizes were reduced for punishment trials (human: M=16, F=16; mouse: M=5, F=9). Only humans had a significant low > high probability theta-band contrast (Table S1, S2, Figure S3). There was a significant main effect of sex in mice on punishment trials (female > male). For the second cohort (100% vs 50%), there was no opportunity to assess expectation in punishment (i.e., no punishers to responses on the 100% correct stimulus), so these analyses were restricted to the first cohort.

|  |  | **Low Freq** | **High Freq** | **Start Time** | **End Time** | ***t*** | **df** | ***p*** | ***d*** | **Match?** |
| --- | --- | --- | --- | --- | --- | --- | --- | --- | --- | --- |
| PLT Pun Theta | Human | 4 | 6.5 | 450 | 750 | 2.33 | 31 | 0.03 | 0.35 | No |
|  | Mouse | 4.5 | 7.5 | 500 | 800 | 0.97 | 12 | 0.36 | 0.17 |  |

**Table S2. Simple effects for punishment conditions on the PLT.** Time and frequency ranges for event-related tf-ROIs, as well as simple effects statistical contrasts for PLT low > high probability punishment.

The prevalence of frontal midline theta effects in humans has been well-detailed, presenting a compelling target for replication in mice. Notably, theta power to punishment is not theorized to specifically reflect punishment prediction error^55^. Rather, it reflects the calculation of surprise in a system that is sensitive to bad events, and can act as a marker of the need for control. This punishment surprise-evoked theta power was reliably observed in humans, yet it was not observed in mice. However, the degree of learning on the task significantly correlated with the low > high probability theta-band contrast to punishments in mice (Supplemental Figure S4), again suggesting promising returns following addressable issues in training and performance. This same relationship was present, but underpowered, for the low > high probability delta-band contrast to rewards.


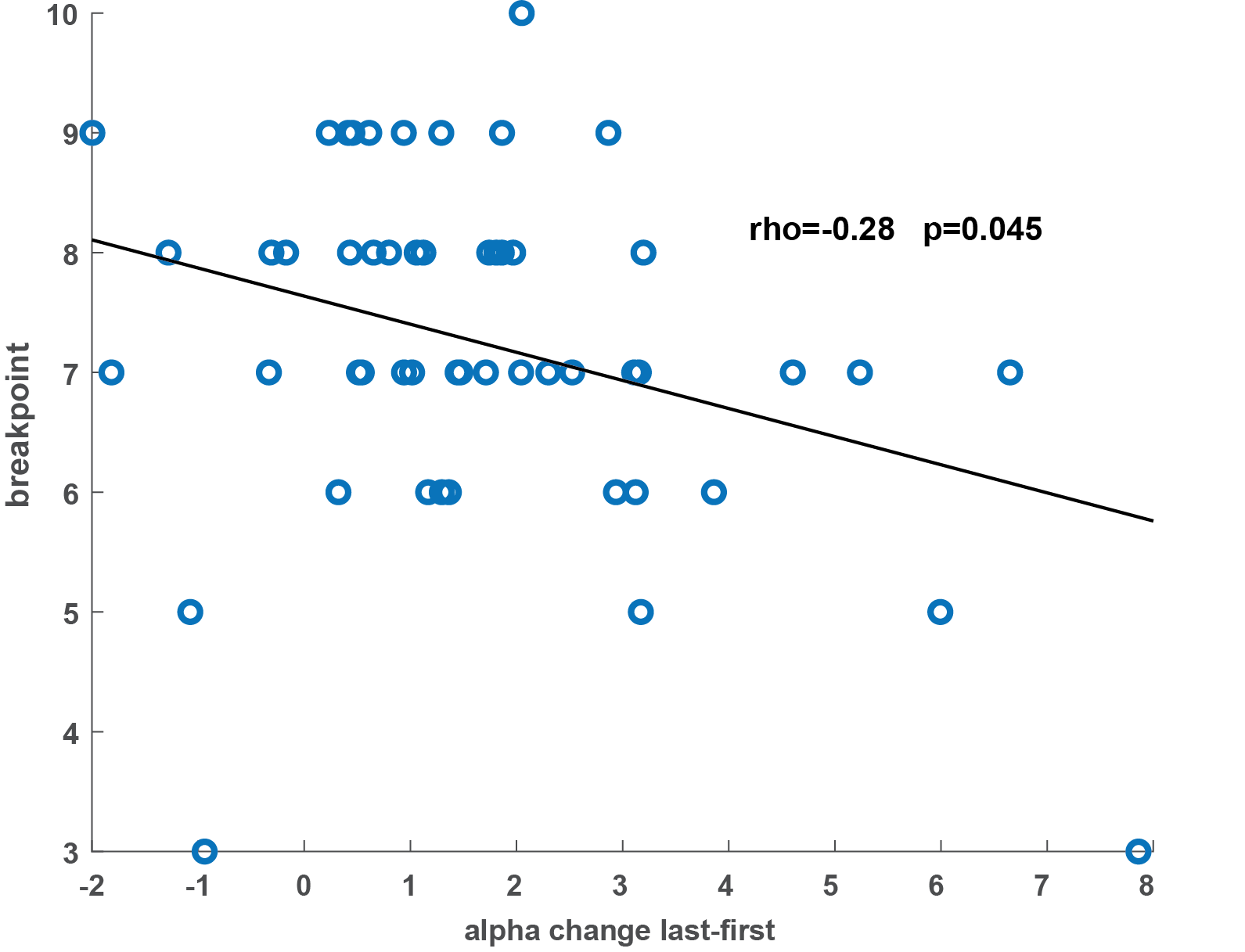


**Figure S2**. **Human PRBT posterior alpha power correlated negatively with breakpoint.** The change in posterior alpha power in humans from last to first trial correlated with the primary outcome measure of motivation, breakpoint. Thus, the higher the motivation, the lower the difference in posterior alpha power.


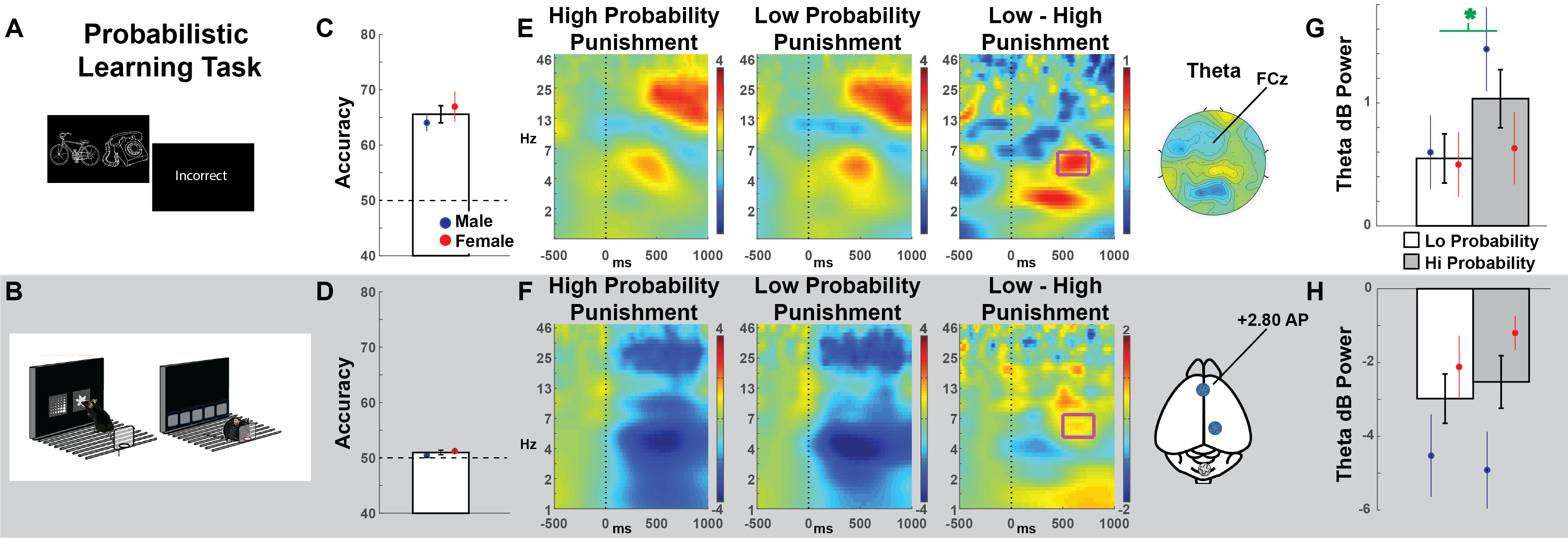


**Figure S3. The Probabilistic Learning Task (PLT) required the subject to select the stimulus that probabilistically led to reward most often.** A-B) In humans and mice, each trial required a choice between two stimulus icons. C-D) Total accuracies for each species, including means split by sex. E-F) Time-frequency plots of expected vs. unexpected punishments at FCz in humans or the anterior lead in mice. The magenta box shows the theta band tf-ROI. G-H) EEG tf-ROI quantification of the unexpected vs. expected punishment difference in frontal theta power. Bars are means (± SEM), green asterisks indicate statistically significant (*p*<0.05) within-subject differences.


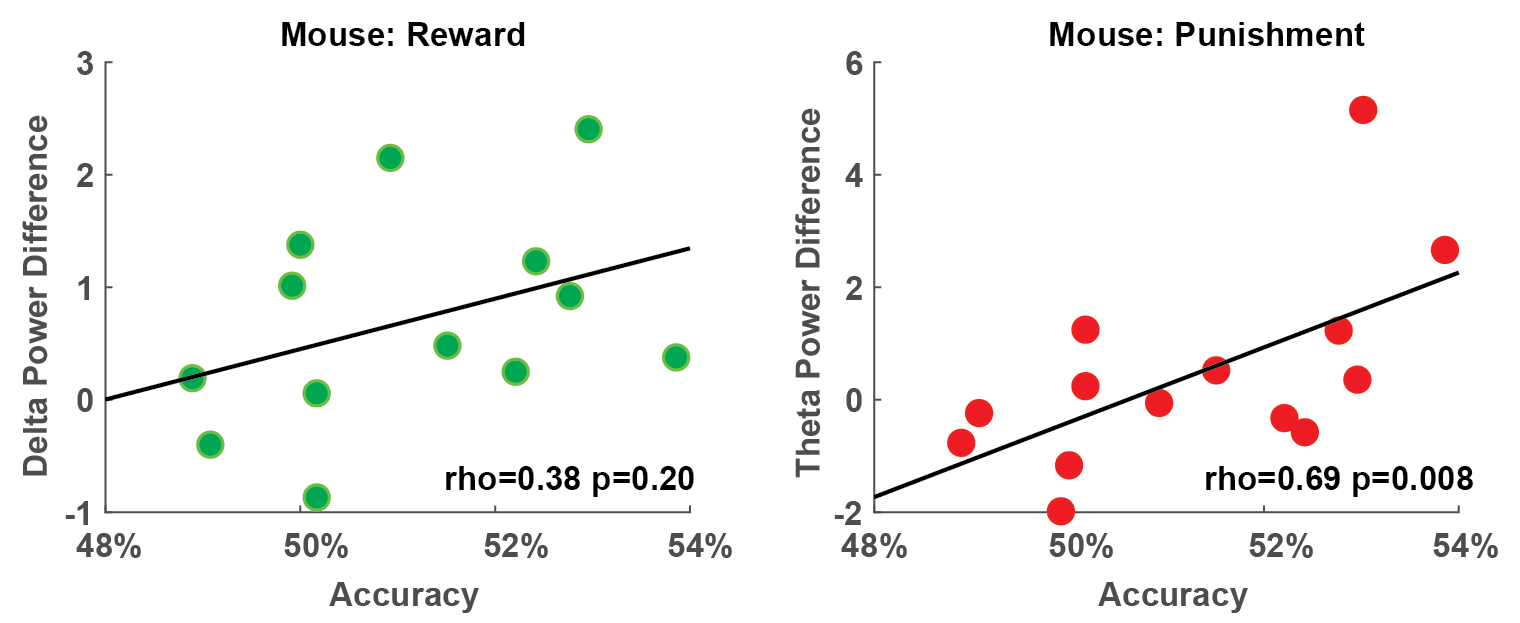


**Figure S4**. **Mouse PLT brain-behavior relationships in cohort 1.** The expectation-related EEG differences should depend on adequate stimulus-action learning. Since mouse performance in the first cohort was only modestly better than chance, we investigated if the size of the surprise-related EEG power change varied with behavioral performance. These correlations indicate that the low > high probability change in theta power to punishers was increasingly observed in mice who performed better on the task. A similar, but less strong relationship was found between low > high probability change in reward-related delta power. These findings bolster the interpretation that these EEG effects should be expected in future cohorts once training has reached adequate discrimination and performance can be maintained.
